# Developing a cluster-based approach for deciphering complexity in individuals with neurodevelopmental differences

**DOI:** 10.3389/fped.2023.1171920

**Published:** 2023-09-18

**Authors:** Tania Cuppens, Manpreet Kaur, Ajay A. Kumar, Julie Shatto, Andy Cheuk-Him Ng, Mickael Leclercq, Marek Z. Reformat, Arnaud Droit, Ian Dunham, François V. Bolduc

**Affiliations:** ^1^ Département de Médecine Moléculaire de L'Université Laval, Centre de Recherche du CHU de Québec-Université Laval, Québec, QC, Canada; ^2^ Department of Pediatric Neurology, University of Alberta, Edmonton, AB, Canada; ^3^ European Molecular Biology Laboratory, European Bioinformatics Institute (EMBL-EBI), Wellcome Genome Campus, Hinxton, United Kingdom; ^4^ Department of Electrical and Computer Engineering, University of Alberta, Edmonton, AB, Canada; ^5^ Department of Medical Genetics, University of Alberta, Edmonton, AB, Canada; ^6^ Neuroscience and Mental Health Institute, University of Alberta, Edmonton, AB, Canada

**Keywords:** neurodevelopmental differences, global developmental delay, phenotype, whole exome sequencing, hierarchical agglomerative clustering, k-means clustering, basket trials

## Abstract

**Objective:**

Individuals with neurodevelopmental disorders such as global developmental delay (GDD) present both genotypic and phenotypic heterogeneity. This diversity has hampered developing of targeted interventions given the relative rarity of each individual genetic etiology. Novel approaches to clinical trials where distinct, but related diseases can be treated by a common drug, known as basket trials, which have shown benefits in oncology but have yet to be used in GDD. Nonetheless, it remains unclear how individuals with GDD could be clustered. Here, we assess two different approaches: agglomerative and divisive clustering.

**Methods:**

Using the largest cohort of individuals with GDD, which is the Deciphering Developmental Disorders (DDD), characterized using a systematic approach, we extracted genotypic and phenotypic information from 6,588 individuals with GDD. We then used a k-means clustering (divisive) and hierarchical agglomerative clustering (HAC) to identify subgroups of individuals. Next, we extracted gene network and molecular function information with regard to the clusters identified by each approach.

**Results:**

HAC based on phenotypes identified in individuals with GDD revealed 16 clusters, each presenting with one dominant phenotype displayed by most individuals in the cluster, along with other minor phenotypes. Among the most common phenotypes reported were delayed speech, absent speech, and seizure. Interestingly, each phenotypic cluster molecularly included several (3–12) gene sub-networks of more closely related genes with diverse molecular function. k-means clustering also segregated individuals harboring those phenotypes, but the genetic pathways identified were different from the ones identified from HAC.

**Conclusion:**

Our study illustrates how divisive (k-means) and agglomerative clustering can be used in order to group individuals with GDD for future basket trials. Moreover, the result of our analysis suggests that phenotypic clusters should be subdivided into molecular sub-networks for an increased likelihood of successful treatment. Finally, a combination of both agglomerative and divisive clustering may be required for developing of a comprehensive treatment.

## 1. Introduction

Neurodevelopmental disorders/difference (NDDs) are a broad group of disabilities characterized by impairments of personal, social, academic, or occupational functioning and/or skill development (1). NDDs affect approximately 18% of the population (2–8) and have a significant impact on the individual, their family, and society (9, 10). Global developmental delay (GDD) is a subtype of NDD and has a prevalence rate of approximately 3% (11, 12). GDD is diagnosed when an individual under the age of 5 years fails to meet the expected developmental milestones in two or more domains of development, such as language, gross or fine motor skills, or social functioning (13).

Individuals with GDD, as in most NDD, present within a spectrum of severity (14). Moreover, several genes have now been linked to GDD (15), but each gene individually affects a relatively small number of individuals, making them known as rare disorders in most cases. The phenotypic complexity, combined with the genetic heterogeneity and rarity, has hampered our ability to translate our understanding of the molecular underpinning of GDD into targeted interventions that are clinically approved and mechanism-based. Most trials conducted previously have been focused on translating candidate drugs identified from pre-clinical investigations on a given gene into individuals with mutation in that gene. Unfortunately, this approach has been accompanied by challenges in recruitment, clinical diversity, and a high number of genes to target.

Fortunately, a novel approach to clinical trials is emerging, known as basket trials (16), which aims at testing candidate drugs in a group of disorders related by shared pathophysiology, consequently improving the cost-effectiveness of the trial. So far, this approach has been proven to be very productive in oncology, where participants with different diagnoses but share a common underlying dysregulated molecular pathway are treated with the same therapeutics (17). In GDD, individuals could be subgrouped based on their phenotypic or genotypic profiles (18). Therefore, it is important to gain a better understanding of how GDD individuals could be clustered.

In general, two approaches have been used in clustering: agglomerative (also referred to as bottom-up) or divisive (referred to as top-down) (19, 20). Hierarchical agglomerative clustering (HAC) aims at identifying homogeneous groups of individuals based on their phenotypic profiles, which does not assume a given number of cluster and therefore can lead to a combination of phenotypes (21, 22). HAC has been widely used due to its ability to detect the natural number of clusters in a dataset (23–25). On the other hand, a divisive approach such as k-means clustering (19) requires a set number of clusters to be established and then assigns individuals to each cluster based on their similarity (26–28).

Here, we show how these two approaches can be applied to clustering individuals in the largest cohort of individuals with GDD: the Deciphering Developmental Disorders (DDD) cohort (29).

## 2. Materials and methods

### 2.1. Cohort description

The data included in the DDD dataset were acquired from 24 clinical genetics centers in the United Kingdom National Health Service and the Republic of Ireland. A total of 13,462 individuals with undiagnosed developmental disorders were included in this study. After obtaining ethics approval at our centers, and with permission from the DDD consortium, we analyzed the dataset for phenotypes of the individuals.

In DDD, the human phenotype ontology (HPO) is used to describe the phenotypic information of the individuals (30). HPO contains over 15,000 terms, which describes phenotypic abnormalities and allows the use of standardized and controlled vocabulary for listing phenotypes (31). We divided the HPO identified in individuals with GDD between structural (dysmorphic features or congenital malformation) and functional (affecting behavior or clinical symptoms). We focused this study on functional HPO, considering our goal to cluster patients for future interventions.

### 2.2. Genomic sequence analysis

The exome sequence data of GDD-phenotyped individuals were analyzed in two stages. In the first stage, the existing GRCh37/hg19 exome sequence was realigned to the GRCh38 genome reference sequence. Then, short variant [single nucleotide variants (SNVs) and indels] calling was performed using the GATK best practices (32), which involves realigning reads to the GRCh38 reference genome, variant calling using the HaplotypeCaller and joint genotyping. Finally, variant quality recalibration and refinement steps were performed, leading to high quality variant callset. In the second stage, these variants were annotated for gene information (Ensembl), frequencies (from gnomAD, ExAC, and internal cohort GDD), and pathogenicity (from CADD, ClinVar, and ClinGen). The annotated set of variants in the callset were filtered for gene information, rare variants having minor allele frequency (MAF) value of ≤1%, impact on the transcript, and pathogenicity. The details of the annotation and filtering criteria can be found in Section 1.1 in the Supplementary Material.

### 2.3. Candidate gene list

We searched PubMed using the keywords intellectual disability (ID) and global developmental delay (GDD), and compiled a list of genes from original research and review papers (33–36). We also used genes listed in databases related to NDDs for diseases and phenotypes, and even included the genome-wide association studies (GWAS) using the same keywords [SysID (37, 38), DisGenet (39, 40), HPO (31, 41), OMIM (42, 43), Orphanet (44), Phenolyzer (45, 46), Ingenuity Pathway Analysis (Qiagen), Open Targets (47, 48), AutDB (49)]. We have added the Intellectual Disability NGS Radboudumc and Fulgent gene panels to achieve the most complete overview. Each gene list was obtained separately, and then only those genes that appeared at least three times in the collected data were retained, resulting in a final list of 2,537 genes (see Supplementary Table 1).

### 2.4. Clustering strategies

#### 2.4.1. Hierarchical agglomerative clustering of phenotypes

Among all the HPO-based phenotypes identified in individuals with GDD, we considered the functional phenotypes (as opposed to morphological features) for this cluster analysis. Since all the phenotypes can be treated as binary features and the dissimilarity between two individuals can be calculated based on their shared and distinct phenotypes, Jaccard distance was used (50) to measure the dissimilarity between the individuals, which is calculated as follows:

$$D(I_{i},I_{j})=1-\;\frac{\left|P_{i}\cap P_{j}\right|}{\left|P_{i}\cup P_{j}\right|}$$

where $D(I_{i},I_{j})$ is the distance between individual $I_{i}$ and $I_{j}$ with set of phenotypes $P_{i}$ and $P_{j}$, respectively. Once the distance matrix representing the Jaccard dissimilarity between all the individuals is obtained, hierarchical clustering with ward linkage method is applied. To assess the cluster validity, we selected the silhouette index (SIL) due to its ability to provide assessment at the cluster and individual level. SIL is an internal measure that considers both the intra-cluster and inter-cluster distances to provide the estimate of compactness and separateness of the clusters (51). For each data point $I_{i}$, the distance to all data points belonging to the same cluster is calculated, and the average of these distances is referred as $I_{j}$. Then, the distance to all data points belonging to other clusters is calculated, and the smallest of them is referred as $b_{i}$. The silhouette coefficient for each individual is calculated as follows:

$$SIL_{i}=\frac{\left(b_{i}-\;a_{i}\right)}{max(a_{i},b_{i})}$$

Overall clustering results can be assessed by taking the average silhouette index of all the individual points, and its value ranges from worst value −1 to best value 1.

### 2.4.2. Dependency model for genes/phenotype clustering

We performed a divisive clustering approach with k-means using a dependency model to cluster the genes associated to phenotypes. The dependency model (Supplementary Figure S1) was created under a probabilistic framework where the relationship between a given set of phenotypes (H) and genes (G) is inferred for the affected individuals (I). This dependency model captures the direct relation among individuals having distinct GDD phenotypes (solid red line in Supplementary Figure S1). Similarly, the same affected individuals carry those rare genetic variants among a set of genes. However, it is difficult to establish whether these genes cause the individuals to acquire GDD or because of inheriting these GDD-related traits, the individuals acquired mutations in these genes. The direction of causality cannot be established between phenotype and genes; hence, these can only be inferred probabilistically (represented by a broken red line in Supplementary Figure S1).

The underlying dependency model facilitates a framework to identify clusters of genes with respect to a given set of phenotypes. Mathematically, this can be represented as computing probability distribution of genes, which is conditional to the phenotypes given by *P*(*G*|*H*):

$$P(G|H)=\overset{N}{\underset{\;j=1}{\sum }}\frac{P(I_{j})P(I_{j})P(I_{j})}{\sum_{\;j=1}^{N}P(I_{j})P(I_{j})}$$

Since our task is to compare probability distribution of genes with respect to a given set of phenotypes, the denominator in the above equation can be ignored, and it can be simplified as follows:

$$P(G|H)\infty \overset{N}{\underset{\;j=1}{\sum }}P(I_{j})P(I_{j})P(I_{j})$$

This eventually yields a matrix of probability distribution where rows represent the genes and columns represent the phenotypes. A k-means clustering was then applied to a subset of the probability matrix, including the phenotypes of interest (52). The elbow and silhouette methods were used to determine the optimal number of clusters (53). To highlight significant differences between phenotypes for each gene group, a *t*-test was applied to the clustering results. We corrected the results obtained using the Bonferroni correction to adjust the *p*-values for multiple comparisons. Adjusted *p*-values less than or equal to 0.05 were considered statistically significant.

### 2.5. Protein–protein interaction and pathway enrichment analysis

The resulting gene clusters were then analyzed by protein–protein interaction prediction, clustering, and pathway enrichment using STRING v11.5 (54). The Markov clustering algorithm (MCL) was used to identify the gene sub-networks (55). The inflation parameter of MCL was set to 1.5 (56). The functional enrichment analysis of each module was also performed in the STRING v11.5 database using the GO terms and REACTOME pathway. The false discovery rate obtained from the functional enrichment analysis describes the degree of significance of the enrichment. The *p*-values are corrected for multiple testing in each category using the Benjamini–Hochberg procedure. Visualizations of protein–protein interactions and clusters were also obtained using STRING v11.5.

## 3. Results

### 3.1. Individuals with GDD present with phenotypic diversity

**Figure 1:**
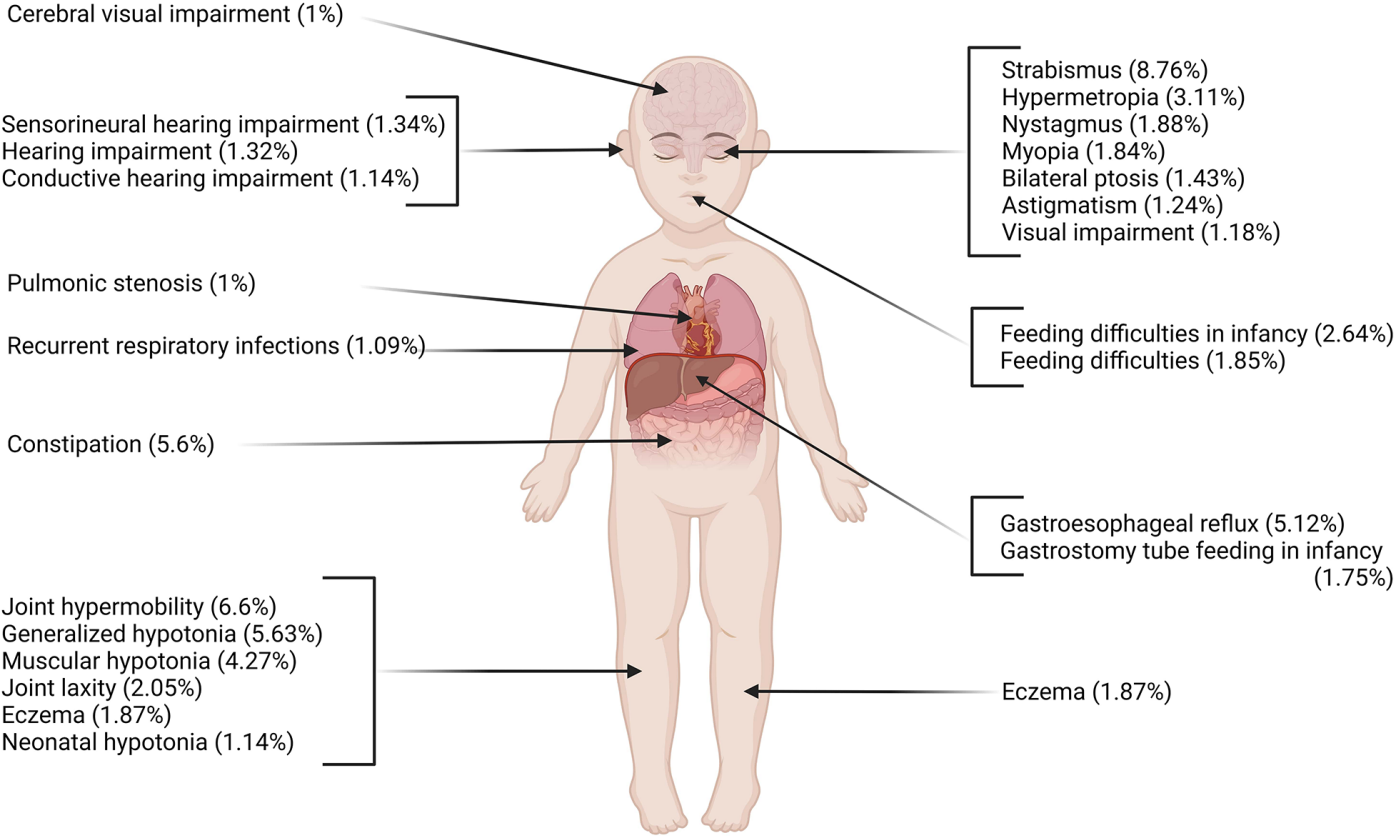
Most prevalent physiological phenotypes in individuals with GDD using HPO ontology (frequency >1%). Multiple systems are affected including neurological, respiratory, gastrointestinal, and locomotor systems. Created with BioRender.com.

Among all participants with NDD in the DDD cohort, the individuals with GDD represent 49.13% (6,588 probands) of the population. We identified 761 physiological phenotypes and 2,158 morphological phenotypes occurring in this cohort. The phenotypes that are most commonly represented (>1%) affected multiple systems (neurological, gastrointestinal, locomotor) in individuals with GDD (Figure 1, Table 1). The physiological phenotypes that are neurodevelopmental-related comprised autistic behavior (6.25%), autism (4.57%), absent speech (AS) (4.36%), aggressive behavior (3.25%), and stereotypy (4.42%) (including 2.34% of the global term, 2.06% recurrent hand flapping, 0.18% repetitive compulsive behavior, and 0.17% tongue thrusting). For other physiological issues, seizures were reported in 21.71% of individuals with GDD (Supplementary Table S2), followed by hypotonia (15.12%), strabismus (8.76%), joint hypermobility (6.60%), and sleep disturbance (4.36%). The most frequent systemic phenotypes involved constipation (5.6%) and gastroesophageal reflux (5.12%).

**Table 1:** Most prevalent (>1%) physiological phenotypes in individuals with GDD.

Physiological phenotype	No. of all GDD	% of all GDD
Neurodevelopmental		
Delayed speech and language development	1,037	15.74
Autistic behavior	412	6.25
Autism	301	4.57
Absent speech	287	4.36
Specific learning disability	173	2.63
Cognitive impairment	121	1.84
Delayed gross motor development	113	1.72
Attention deficit hyperactivity disorder	106	1.61
Developmental regression	105	1.59
Motor delay	91	1.38
Intellectual disability, moderate	74	1.12
Intellectual disability	69	1.05
Intellectual disability, severe	68	1.03
Other neurological		
Seizure	796	12.08
Sleep disturbance	287	4.36
Aggressive behavior	214	3.25
Generalized-onset seizure	178	2.70
Drooling	167	2.53
Stereotypy	154	2.34
Behavioral abnormality	145	2.20
Recurrent hand flapping	136	2.06
Generalized non-motor (absence) seizure	121	1.84
Central hypotonia	113	1.72
Bilateral tonic-clonic seizure	108	1.64
Gait ataxia	107	1.62
Epileptic spasm	93	1.41
Broad-based gait	87	1.32
Febrile seizure (within the age range of 3 months to 6 years)	81	1.23
Generalized myoclonic seizure	75	1.14
Impaired social interactions	73	1.11
Abnormal aggressive, impulsive, or violent behavior	69	1.05
Ataxia	69	1.05
Short attention span	69	1.05
Dysphagia	67	1.02
Eyes		
Strabismus	577	8.76
Hypermetropia	205	3.11
Nystagmus	124	1.88
Myopia	121	1.84
Bilateral ptosis	94	1.43
Astigmatism	82	1.24
Visual impairment	78	1.18
Cerebral visual impairment	66	1.00
Ears		
Sensorineural hearing impairment	88	1.34
Hearing impairment	87	1.32
Conductive hearing impairment	75	1.14
Cardiovascular system		
Pulmonic stenosis	66	1.00
Respiratory system		
Recurrent respiratory infections	72	1.09
Digestive system		
Constipation	369	5.60
Gastroesophageal reflux	337	5.12
Feeding difficulties in infancy	174	2.64
Feeding difficulties	122	1.85
Gastrostomy tube feeding in infancy	115	1.75
Other		
Joint hypermobility	435	6.60
Generalized hypotonia	371	5.63
Muscular hypotonia	281	4.27
Joint laxity	135	2.05
Eczema	123	1.87
Neonatal hypotonia	75	1.14

We present the number of individuals with GDD from the DDD dataset presenting with the phenotypes listed. The phenotypes were structured as either neurodevelopmental, other neurological, or by target organs outside the brain.

We also delineated the morphological features observed most frequently in individuals with GDD (Supplementary Table S3). With regard to growth parameters, microcephaly was found in 19.74% of individuals. Macrocephaly was much less common in GDD (5.53%). Short stature was also frequently reported (5.83%). Cardiac malformations were the most commonly encountered malformation with a prevalence rate of 7.14%. Scoliosis was present in 2.58% of individuals.

### 3.2. Genotypic diversity in individuals with GDD

**Figure 2:**
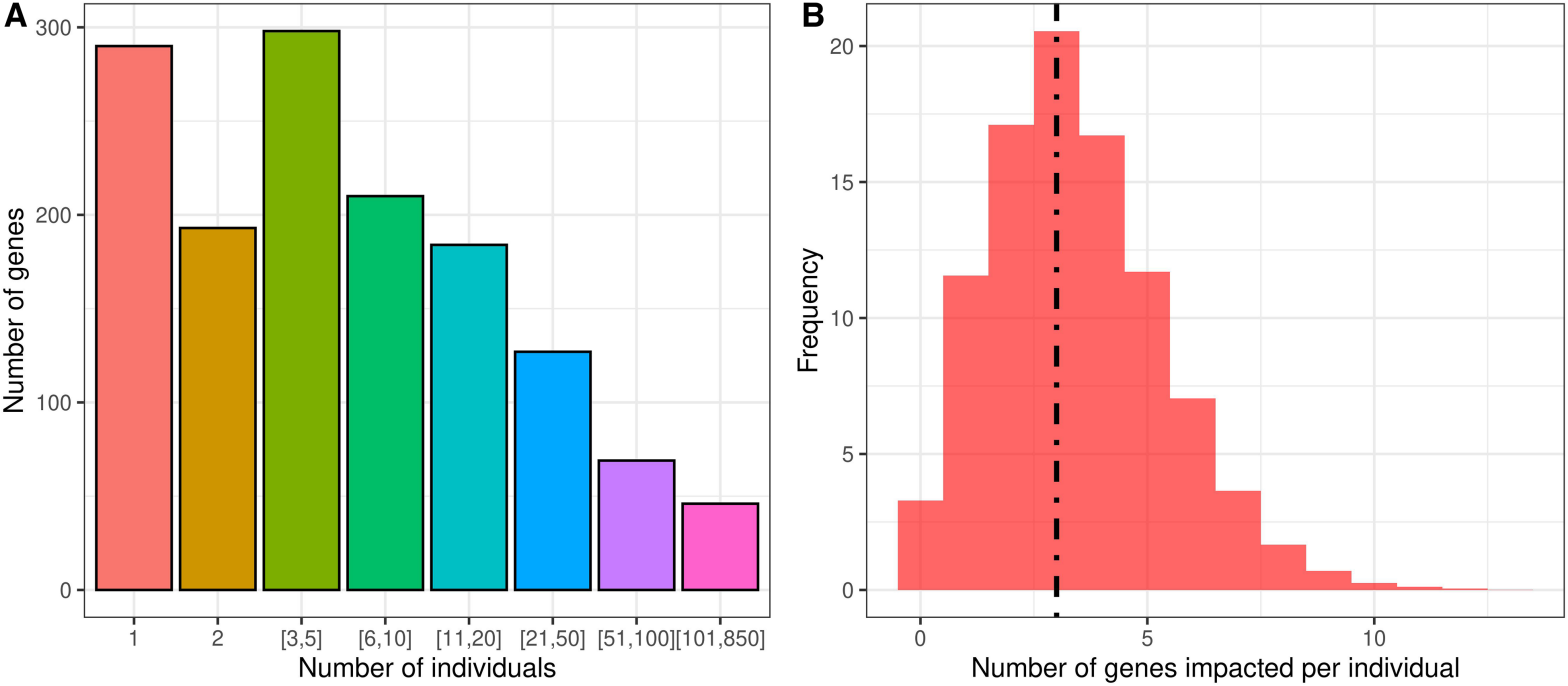
Analysis of the overall cohort of individuals with GDD from DDD reveals the genotypic complexity of GDD. (**A**) Individual GDD/ID candidate genes are rare, as observed clinically. Using the DDD cohort allows us to quantify this with only one to five individuals in a cohort of 6,588 individuals with GDD. (**B**) Individuals with GDD present most of the time with multiple pathogenic variants in distinct genes. The dotted line refers to the median at three genes per individual.

We identified likely pathogenic and pathogenic variants in the individuals with GDD using ClinVar (57). From our list of 2,537 candidate causal genes, we found that 1,416 genes were affected by pathogenic or likely pathogenic variants in individuals with GDD in the DDD cohort. We found that most genes contained pathogenic variants in only a small number of individuals, suggesting an important genotypic diversity (Figure 2, Supplementary Table S1). Indeed, only 86 genes were found in more than 1% of individuals. We also looked at how many genes from our list were affected by pathogenic/likely pathogenic variants per individual in the GDD cohort. A total of 3.5% of the GDD cohort has no pathogenic/likely pathogenic variants from our gene list, and more than 20% of individuals have three mutated genes (Figure 2).

### 3.3. Comparison of clustering approaches

Next, we compared two approaches of clustering: (1) hierarchical agglomerative where clustering of individuals was based on phenotypes, and (2) where individuals with and without a phenotype are compared for their genetic mutations. For each approach, we identified the genes (from our curated list of GDD/ID genes) harboring likely pathogenic or pathogenic variants (ClinVar).

**Figure 3:**
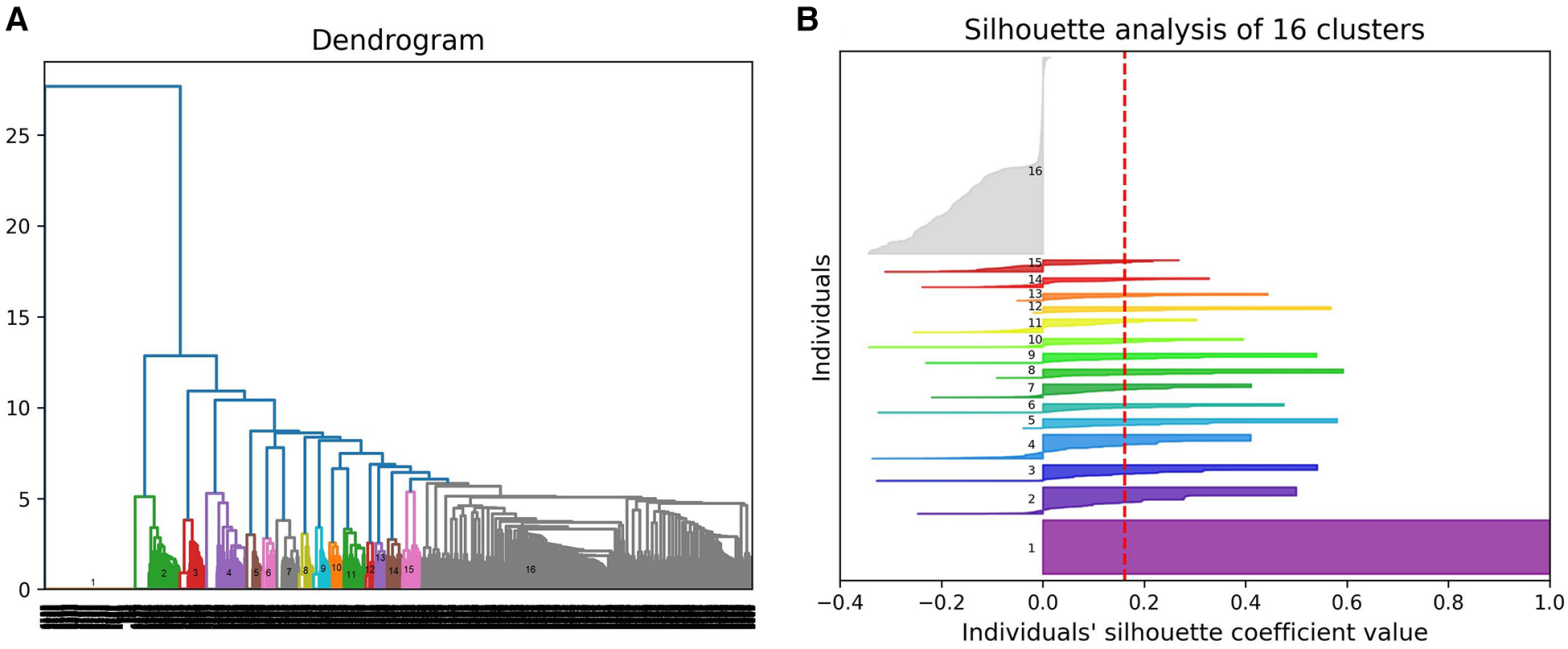
Hierarchical agglomerative clustering using phenotypic profiles of individuals with GDD. (**A**) Dendrogram presenting 16 clusters of individuals. (**B**) Silhouette plot for all the individuals for cluster validity analysis.

**Figure 4:**
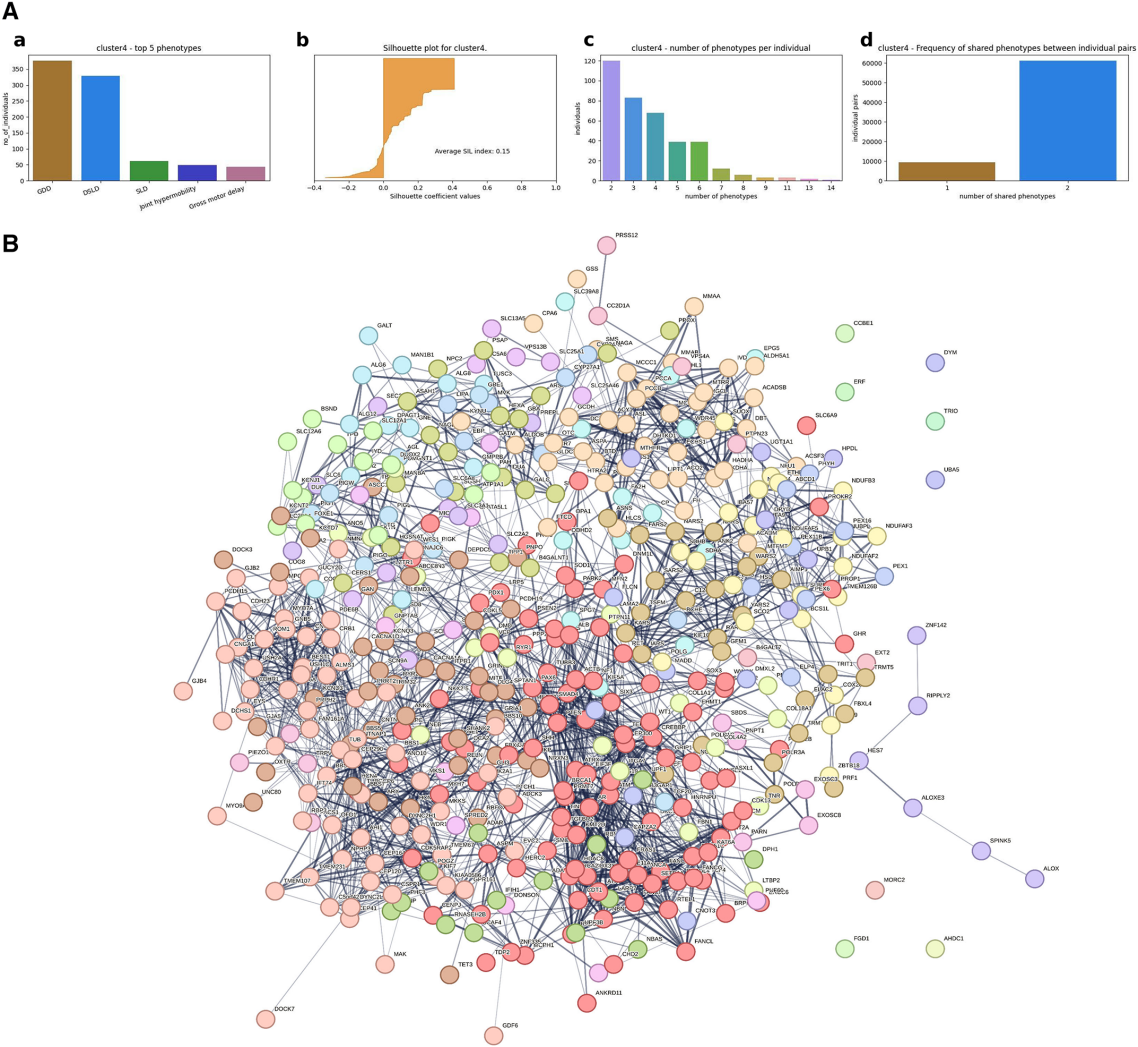
Gene network from individuals with GDD + DSLD using hierarchical agglomerative clustering (Cluster 4). (**A**) Cluster level analysis of the hierarchical clustering results. (a) Top five phenotypes among the individuals. (b) Silhouette score for all the individuals and the average. (c) Distribution of number of phenotypes per individual. (d) Distribution of shared number of phenotypes among all individual pairs. For Cluster 4, DSLD is the dominant phenotype. With some individuals having negative silhouette index, overall cluster level index is positive, indicating that most of the individuals are in the right cluster. Similar to Cluster 2, the majority of the individuals have two phenotypes, but the number of phenotypes per individual ranges up to 14, indicating the phenotypic variability among individuals. (**B**) Overall representation. Each color corresponds to a representative sub-network of more closely related genes.

**Figure 5:**
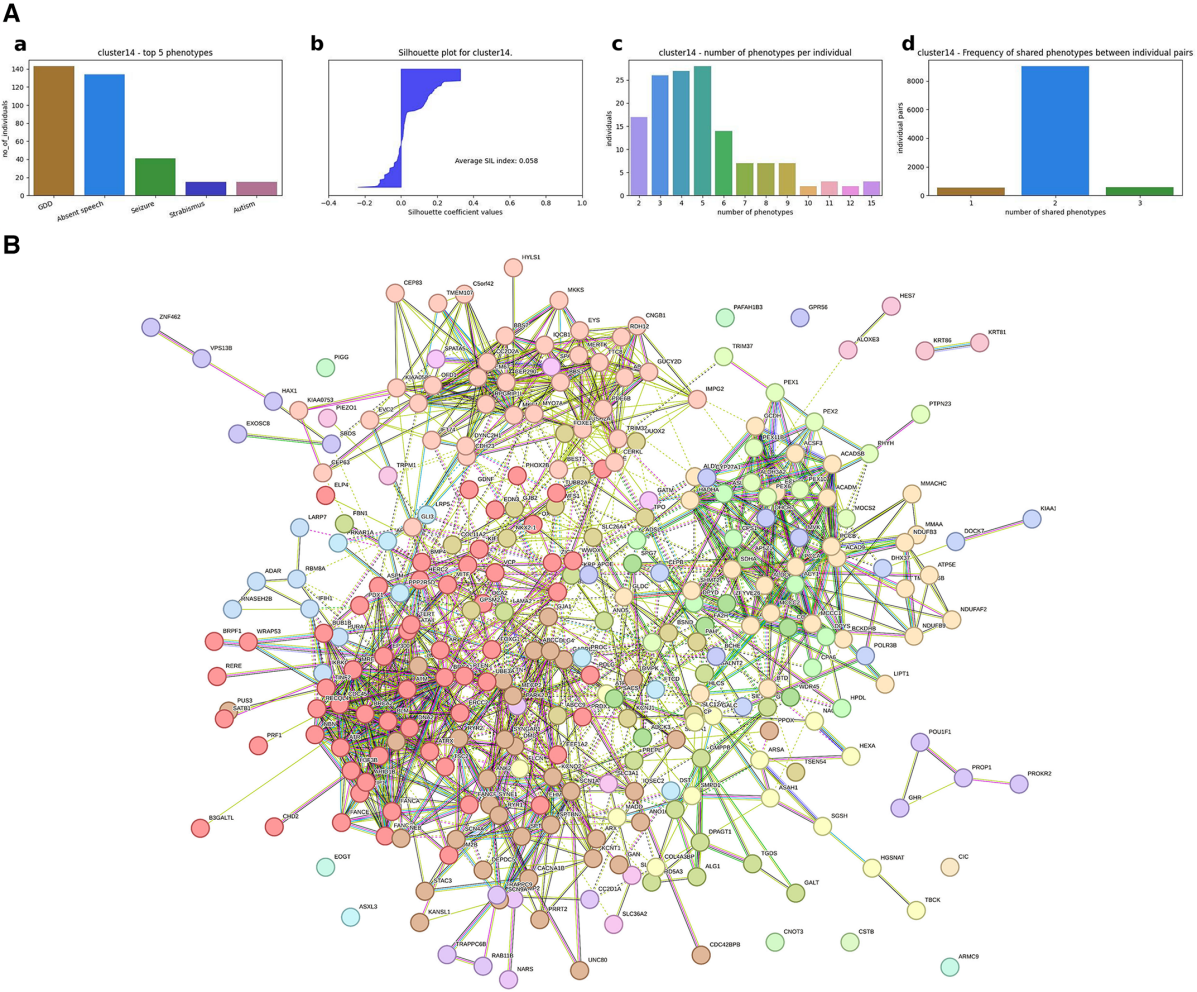
Gene network for individuals with AS identified by hierarchical agglomerative clustering (Cluster #14). (**A**) (a) Top five phenotypes among the individuals. (b) Silhouette score for all the individuals and the average. (c) Distribution of number of phenotypes per individual. (d) Distribution of shared number of phenotypes among all individual pairs. For Cluster 14, absent speech is the dominating phenotype, and the average silhouette index for the cluster is 0.058, which is slightly above zero. Almost half of the individuals have a negative SIL index, which could possibly be due to the high variability in the number of phenotypes per individual. As shown in the third plot for Cluster 14, the number of phenotypes per individual ranges up to 15, and the majority of the individuals share only two phenotypes, leading to less similarity among individuals. (**B**) Overall representation. Each color corresponds to a representative sub-network of more closely related genes.

**Figure 6:**
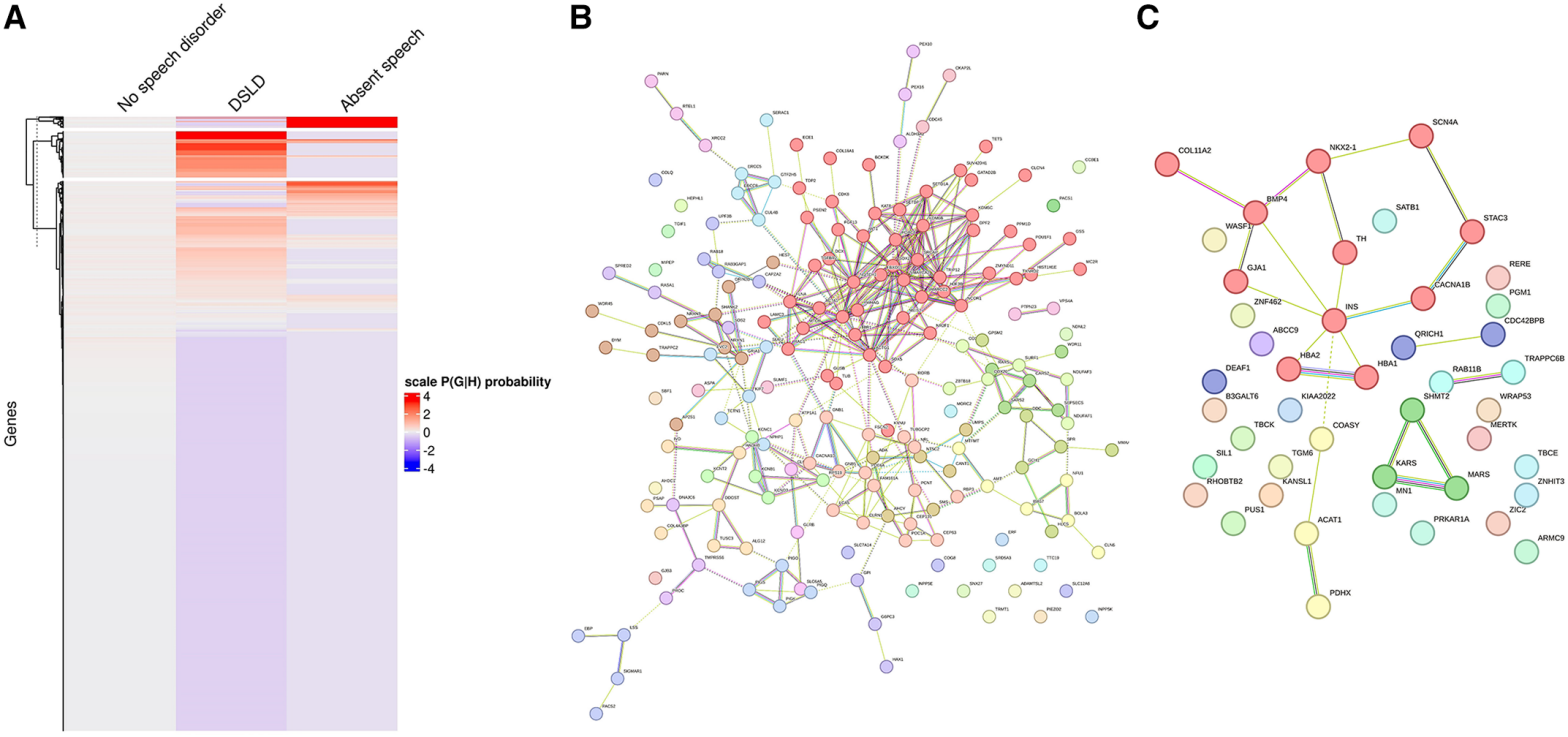
Gene network for individuals with DSLD or AS identified from k-means clustering. (**A**) k-means clustering of individuals without speech disorder, delayed speech, or absent speech. (**B**) Overall gene network for individuals with delayed speech. (**C**) Overall gene network for AS. Each color corresponds to a representative sub-network of more closely related genes.

(1) A hierarchical agglomerative clustering of all individuals with GDD resulted in 16 distinct clusters (as visualized in the dendrogram in Figure 3A). The silhouette index of all the individuals is displayed in Figure 3B. In order to validate the clustering results, we assessed each cluster separately. Except for cluster 16, all other clusters had a positive silhouette index for majority of the individuals, indicating that the individuals are clustered in the correct group. However, in each cluster, some individuals had a negative silhouette index. The variability in the number of phenotypes per individual and fewer shared phenotypes among individuals could possibly lead to the distortion in the coherence of the clusters (detailed analysis in Supplementary Figure S2). While we observed more than two phenotypes per cluster, most individuals presented with GDD + 1 dominant phenotype. Cluster 16, containing 3,077 individuals, showed no dominant phenotype apart from the GDD.
  We focused on clusters related to speech since 20% of individuals from the GDD cohort presented with delayed speech to various degrees. Milder defects were categorized as delayed speech, which was present in 15.74% of individuals with GDD, and more severe defects were categorized as absent speech, present in 4.36% of individuals. HAC Cluster 4 was characterized by delayed speech and language development (DSLD), which presented in 329 out of 376 individuals in this cluster, while Cluster 14 defined individuals with absent speech, which presented in134 out of 143 individuals in this cluster (Figures 4A, 5A). Cluster 4 comprised 376 individuals with likely pathogenic or pathogenic variants in 483 candidate genes, while Cluster 14 comprised 143 individuals with likely pathogenic or pathogenic variants in 303 candidate genes. We then identified a gene network for each cluster (Figures 4B, 5B). We observed that in both networks, there were sub-networks (of more closely related genes with different significantly enriched molecular functions). For DSLD, we identified 27 sub-networks, with 12 of these sub-networks containing more than 10 genes with enrichment in the following pathways: sub-network 1: DNA repair, cell cycle, transcription, meiosis, and regulation of TP53 activity; sub-network 2: cilium assembly, visual photo transmission, cargo trafficking, and Hedgehog signaling; and sub-network 3: cation channel complex, channel activity, and plasma membrane complex (see Supplementary Table S4 for all sub-networks). For AS, we identified 23 subclusters, with eight of these containing more than 10 genes with enrichment in the following pathways: sub-network 1: DNA repair, cell cycle, and transcriptional regulation; sub-network 2: cilium assembly, anchoring to the basal membrane; and sub-network 3: interaction between LI and ankyrin, L1CAM (see Supplementary Table S5 for all sub-networks). This overlap in gene function enrichment between DSLD and AS was also associated with 31.7% overlap in genes identified by HAC for each condition (Supplementary Figure S3A). Interestingly, those genes showed enrichment in more specific pathways associated previously to cognition (58), such as signal transduction and secondary messenger, transcription, and chromatin modification (Supplementary Table S6).
(2) Next, using the dependency probabilistic models, we determined that three clusters would be optimal for k-means clustering to see if we could identify subgroups of individuals with speech defects. First, we noted that k-means clustering separated individuals with DSLD and AS with no gene overlap (Supplementary Figure S3B). As in HAC, k-means clusters subdivided at the gene network level (Figure 6). DSLD had three sub-networks containing more than 10 genes, and there were 24 sub-networks in total; the enrichment was based on chromatin-modifying enzyme, signal transduction, NOTCH signaling (sub-network 1); visual phototransduction, recruitment of mitotic centrosomes, noradrenaline inhibition of insulin, calcium pathway (sub-network 2); and protein interaction at the synapse, neurexin and neuregulin, trafficking of GluR2-containing AMPA receptors (sub-network 3) (see Supplementary Table S7 for all sub-networks). In AS, only the sub-network 1 had more than 10 genes: oxygen binding (subcluster 1), ribonucleoside synthetic process (subcluster 2), and amino acid binding (subcluster 3) (see Supplementary Table S8).

Next, we assessed if genes that were identified for DSLD and AS overlapped between HAC and k-means clusters. Surprisingly, only 12.7% and 8.4% of genes overlapped for DSLD and AS between the two clustering approaches, respectively (Supplementary Figure S4).

**Figure 7:**
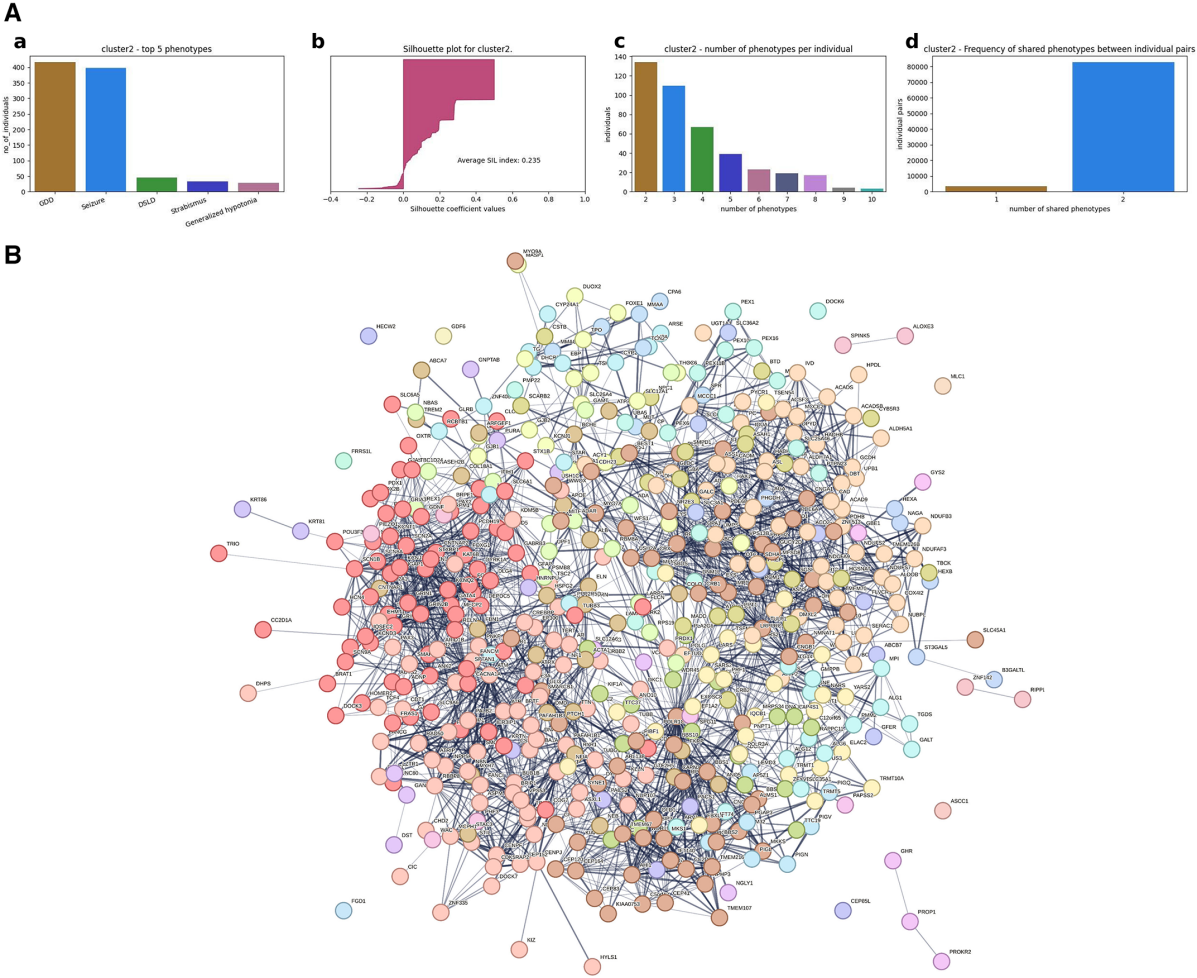
Genetic makeup and clustering for individuals with GDD and seizure (Cluster #2). (**A**) (a) Top five phenotypes among the individuals. (b) Silhouette score for all the individuals and the average. (c) Distribution of number of phenotypes per individual. (d) Distribution of shared number of phenotypes among all individual pairs. For instance, for Cluster 2 in row 2, the first plot shows that seizure is the most dominant phenotype; the second plot shows the silhouette index of all individuals, which is positive for the majority of the individuals, indicating that the individuals are grouped in the right cluster. The third plot in row 2 shows that the majority of the individuals in Cluster 2 has two phenotypes but also ranges up to 10 phenotypes for some of the individuals. The fourth plot shows that most of the individuals share two phenotypes in this cluster. (**B**) Overall gene network for all individuals included in the agglomerative cluster.

In order to assess if these differences in results between clustering approaches were phenotype-specific, we verified if the phenotype seizure, which is commonly associated with GDD, would provide similar results. In HAC, Cluster #2 is characterized by GDD + seizure and pathogenic/likely pathogenic variants in 510 candidate genes (Figure 7A). Again, at the gene network level, we observed fragmentation into sub-networks (Figure 7B, Supplementary Table S9). The MCL clustering found 30 sub-networks, and 13 of them contained more than 10 genes. Each sub-network involved distinct pathways: neuronal system, L1 and ankyrin interaction, axon guidance, synaptic transmission (sub-network 1); transcription regulation, cell cycle, and DNA repair (subcluster 2); and sensory function, cilium, vision, and RNA polymerase (subcluster 3) (see Supplementary Table S9 for all sub-networks).

**Figure 8:**
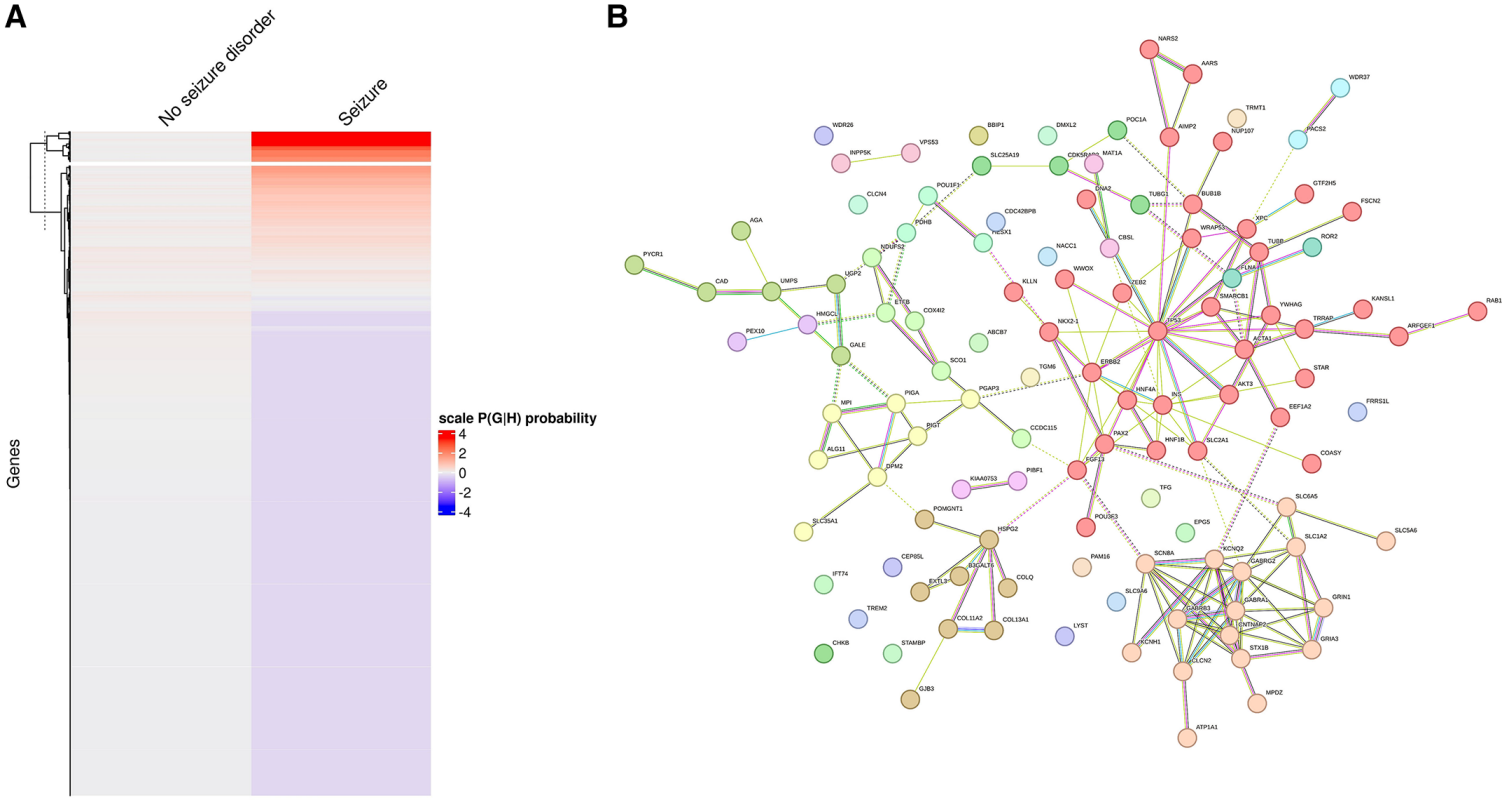
Identification of individuals with/without seizure based on probabilistic genotype–phenotype clustering. (**A**) Clustering of genes based on the association with seizure (on the left are genes not encountered in individuals with GDD and seizure, while on the right are genes associated with GDD and seizure. Higher probability is marked in red). (**B**) Genetic network showing clustering of genes found in individuals with seizures, revealing the presence of sub-networks.

The optimal number of k-means clustering for individuals with GDD with or without seizure was set at two so that only one gene cluster was identified as being related to seizures (Figure 8A). The gene network can also be divided into subgroups, and MCL clustering has highlighted 14 subclusters with only two subclusters having more than 10 genes (Figure 8B). The molecular functions for each cluster were as follows: subcluster 1: organic and hetero-cyclic compound binding and transcription regulator activity; subcluster 2: synaptic function and ion channel and transporter activity; and subcluster 3: extracellular matrix and glycoprotein (see Supplementary Table S10 for all sub-networks).

We then compared the overlap between the two clustering approaches. We found that 62 genes (11%) overlapped between HAC and k-means (Supplementary Figure S5). Importantly, this degree of overlap was similar to the overlap identified between the two clustering approaches when assessing DSLD and AS phenotypes.

## 4. Discussion

Global developmental delay is a clinical entity encountered commonly in both general and specialized medical practice, affecting the lives of approximately 3% of the pediatric population or almost 60 million children worldwide (59). While some individuals with syndromes such as Down, Fragile X, Rett, and Angelman have been diagnosed via targeted gene testing, most individuals remained undiagnosed until the development of genome-wide tools such as chromosomal microarray (CMA) and whole exome sequencing (WES) (15). Together, these genome-wide tools have raised the diagnostic yield in GDD to approximately 60% (15), and have identified approximately 2,000 genes replicated in at least three studies (Supplementary Table S1). This genetic complexity has led to challenging targeted interventions. Novel approaches such as basket trials, where one drug is used to treat distinct but related conditions, have shown the promises of precision medicine (15–17) for those with GDD, but the best approach to clustering individuals remains unknown.

We leveraged the largest dataset of individuals with GDD, which is the DDD (29, 30), and tested various clustering approaches. By including 6,588 individuals with GDD, we were able to identify clear clusters based on phenotypic proximity. We considered the possibility of combining similar or closely related categories into a single main category. However, we decided to maintain the specificity of each HPO term in order to avoid bias in the analyses. Individuals were annotated by their clinician, and we wanted to take advantage of the opportunity to accurately capture the various manifestations of GDD. This accurate phenotypic characterization has enhanced the robustness of our clustering analysis and has facilitated the identification of relevant gene clusters. In our study, we also wanted to use a pragmatic approach, reproducing what would be done when planning a pharmacological intervention in individuals with GDD, so that individuals would be included based on their GDD genetic diagnosis, and not based on a phenotype-specific genotype; hence, the focus on their GDD gene diagnosis.

Since there is an interest (and need due to their high number of genes and low individual prevalence) in combining individuals with GDD into a “basket” targeted by the same drug (one drug-multiple targets), we wanted to assess how this could be achieved. We clustered based on phenotype, following two of the most common approach: divisive based on presence or absence of a phenotype or agglomerative, which does not assume a given number of clusters and therefore can lead to a combination of phenotypes. When comparing clustering approaches, we found that hierarchical agglomerative clustering, an approach where individuals sharing features are grouped together, could identify bigger clusters of genes, but was less precise in segregating genes between related conditions (for instance, delayed speech and language development compared with absent speech). On the other hand, k-means clustering provided more distinct groups of genes but identified fewer genes per phenotype. Importantly, the two methods showed overlap in approximately 10% of the genes they identified. There is a limited overlap in the clusters identified between the two methods. We postulate that this relates to the difference in approach (divisive vs. agglomerative). In the divisive approach, the groups are divided based on the presence or absence of a specific phenotype. We think that this approach leads to a smaller but more stringent set of causative genes. The agglomerative approach identifies phenotypic clusters with a predominant phenotype (but with the inclusion of other phenotypes as well. This may lead to the identification of a different genetic makeup (genes with pleiotropic effect for instance). We believe that our work will point out that the method used to identify individuals in a future treatment trial using a basket trial approach should consider how participants are grouped. Also, for both approaches of clustering, our results have shown consistently that the genetic makeup of a relatively homogenous phenotypic cluster is constituted of multiple subclusters. So for a given basket, it may be possible to understand the response based on that molecular information. This also shows the importance of performing such genetic characterization. It is interesting to observe that while the pathways found in each approach are overall similar, the number of individuals and their individual genes in each cluster are somehow different, which is probably due to the “phenotypic purity” of the cluster.

An important observation in considering future basket trials was that individuals with GDD harboring the same phenotypes could be further subdivided based on genomic information into gene network clusters. This is important due to the reason that individuals within a given basket for a trial may need to be guided by genetic information and assigned to different treatment regimen.

These findings also extended to other common comorbidities such as seizure. Seizures were found to be present in 12.08% of the individuals with GDD. This finding is similar to what has been reported in autism spectrum disorder (ASD) (60), but lower than the 56% prevalence rate of epilepsy in a GDD cohort that was reported recently (61). Importantly, we observed a similar behavior of both clustering approaches: HAC identified more genes than the k-means, and that only approximately 12% of genes overlapped between methods.

It is also important to note that we have made two major filters in this study: first, filter on a list of candidate genes for cognitive neuro difference (GDD and ID), and second to select only the variants in these genes annotated as pathogenic/likely pathogenic in ClinVar. Therefore, it was expected that the enrichment found for each cluster, especially for those found with the HAC method (which shows more genes than the k-means method), would be related to the properties of the selected set and not necessarily cluster specific. In contrast, the number of genes enriched in particular molecular functions changes between clusters; for example, in Cluster 4 (DSLD), DNA binding and transcription stand out in the first subclusters of the 1,417 ID + GDD gene set, but the channel activity stands out far in the subclusters of the 1,417 gene set. This may suggest that ion channels are more related to DSLD.

Overall, our study provides a rationale for the possibility of having success with basket trials in the future for drug development in GDD by showing how large groups of individuals with GDD could be separated into closely related subgroups. It also shows how different clustering approaches will influence the size and nature of the cluster. Furthermore, despite showing shared genetic function, each sub-network (as opposed to the phenotypic cluster) may need to be considered in terms of druggability and potential side effects (for DNA or RNA binding). This highlights the potential importance of genomic sequencing in pharmaceutical trials. Our findings point to the fact that it is important to correlate phenotype with not only a single gene but also take into account the polygenic nature of each individual. It will be important to understand that phenotypes may not be explainable by considering a single gene correlation but rather a polygenic approach and that future work (probably with large sample size) will be required to assess the correlation between combination of pathogenic variants and phenotypic presentation. Also, analysis of each mutation present against the proposed treatment would be important in future clinical trials.

It should be noted that the progress in clustering includes the application of deep learning-based methods, which could potentially complement our research into the genetic basis of GDD. Furthermore, our future research will be based on the assessment of persons with ASD, given the features that they share with GDD. By extending our clustering methods to ASD, we could not only highlight common genetic factors but also refine targeted interventions, broadening the impact of our study beyond GDD. Aware of the role of intronic variants, it will be necessary to integrate whole genome sequencing (WGS) to take into account the whole genetic background of GDD.

Future *in vivo* study will be needed to validate which method is most useful at finding successfully treated clusters. Indeed, it will be important to use animal models this time and patient-derived cell lines to validate the response to candidate treatment for genes belonging to a given cluster. Together, this clustering has the potential to accelerate access to targeted treatment for individuals with GDD.

## Data Availability

Publicly available datasets were analyzed in this study. These data can be found here: https://www.ddduk.org/access.html and https://ega-archive.org/studies/EGAS00001000775: EGAD00001004390 EGAD00001004388.
