# Supplementary material for: Developing a cluster-based approach for deciphering complexity in individuals with neurodevelopmental differences
Source: Front Pediatr. 2023 Sep 18;11:1171920. doi: 10.3389/fped.2023.1171920 (PMC10543689; doi:10.3389/fped.2023.1171920)
Supplement: Supplementary file 11 [file Datasheet1.pdf]

# Supplementary Material

## SUPPLEMENTARY METHODS

### 1.1. Genomic sequence analysis

The exome data pertaining to GDD individuals from DDD were aligned to the GRCh37/hg19 built reference genome. We reprocessed the genomic sequences and re-aligned them on the GRCh38/hg38 reference genome. Short genomic variants which include single nucleotide variants (SNVs) and indels were called using germline variant calling pipeline by implementing the best practices from GATK. The following figure presents the overall workflow which comprises two steps: (a) variant calling and (b) annotation and filtering.

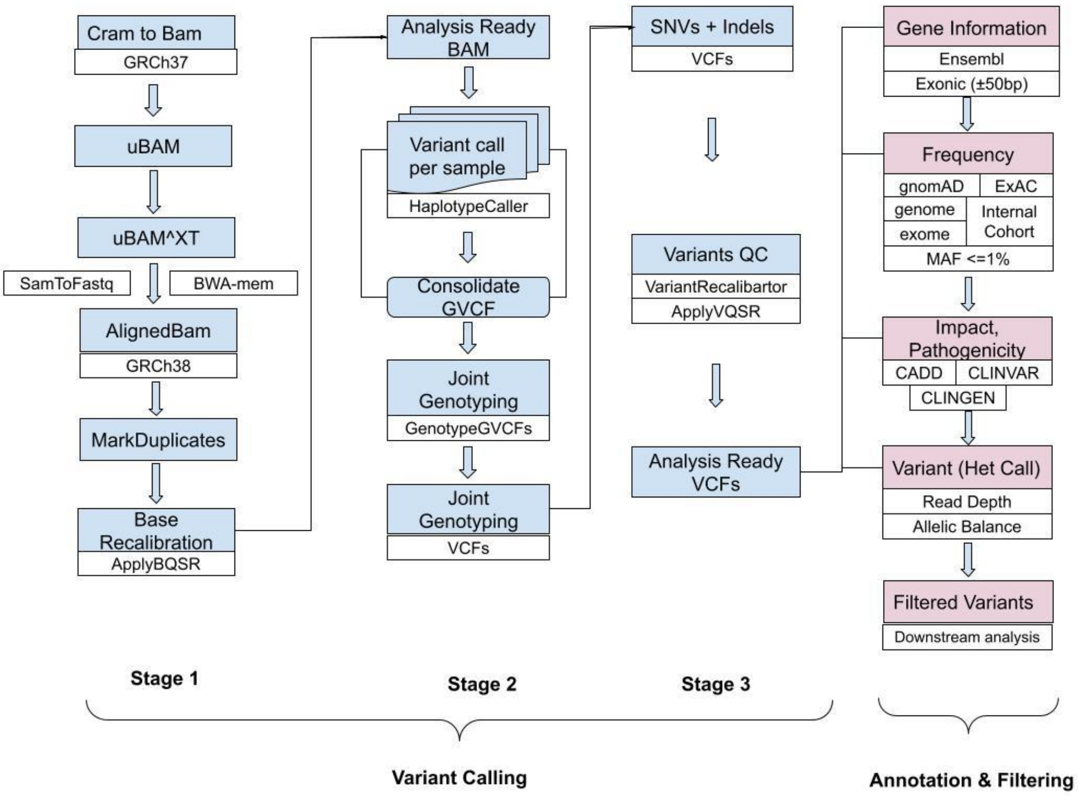

**Workflow for joint variant calling, annotation and filtering.** The first part (colored in blue) of the workflow represents the variant calling. It has three substages where in stage 1 the GRCh37/hg19 aligned BAM files are aligned to the GRCh38/hg19 reference genome. In the second stage, the variants are called per sample and then jointly genotyped for all the samples in the cohort. Finally, in the third stage, the joint genotyped variants in the callset are subjected to filtering and recalibration based on quality. In the second part (coloured in pink), the annotation and filtering strategies are applied. The quality enriched variants for the callset are annotated for information related to gene position, population level frequency, impact and pathogenicity.

The variant calling step has further three stages: (1) alignment of GRCh37/hg19 samples to GRCh38/hg38 reference built and aligned reads are de-duplicated and recalibrated using GATK based tools. (2) The GRCh38/hg38 aligned samples are subject for variant calling using GATK-HaplotypeCaller for the exonic regions (±250 bp) present in the target enrichment file. Post variant calling step, for all these samples (for the given cohort) were jointly genotyped using GATK-GvcfGenotyper in cohort mode. Finally, in (3) the jointly genotyped variant callset were subjected to filtering, annotation and variant quality recalibration using GATK based tools VariantRecalibrator and ApplyVQSR.

The second step of the workflow comprises annotation and filtering of all the variants across all the samples present in the cohort. The variants were annotated for gene information (obtained from Ensembl release 100), frequency present in population databases such as gnomAD (genome-v3.0,v2.1.1, exome - v2.1.1), ExAC (liftover GRCh37/hg19) and GDD internal cohort and pathogenicity scores from databases such as CADD (v1.6), Clinvar/Clingen (release 26/10/2021) and dbNSFP (v4.2.a, downloaded 06/04/2021) which comprises prediction scores from LRT, FATHMM, PROVEAN and MutationTaster, REVEL (v1.3, downloaded on 04/011/2021). The annotation of the variants in the callset were annotated using the Ensembl-VEP program incorporating plugins pertaining to all the above annotation sources.

Finally, the annotated variants are filtered for following criteria:

(1) Filtering out variants that fall outside the exonic regions (±50bp).

(2) MAF <=1% in gnomAD genome (v3) OR gnomADg (v2.1.1) OR gnomADe(v.2.1.1) OR ExAC

(3) Keeping variants with transcript having impact: MODERATE or HIGH OR splice_donor_5th_base_variant OR splice_donor_0th_base_variant OR splice_donor_region_variant OR splice_polyprimidine_tract_variant OR CLNSIG

(4) Transcript prioritization based on variants falling in protein_coding region, having HIGH OR MODERATE effect impact, canonical rank and transcript length.

(5) Variants were filtered for pathogenic/likely_pathogenic in CLINVAR/CLINSEG.

(6) Pertaining to quality, the variants per individual were filtered for

   (a) Read Depth >=5

   (b) Heterozygous calls having allelic balance [0.15,]

### 1.2. Intellectual Disability / Global Developmental Delay gene list

The last update of the gene list is 9 December 2022 for all the databases (SysID, DisGenet, HPO, OMIM, Orphanet, Phenolyzer, Open Targets, AutDB)

SysID : latest update on November 18th 2021
DisGenet : version 7.0, January 2020
HPO: 2022-10-05 Release
OMIM: 2022-12-09 Release
Orphanet: 2022-11-28 Release
Phenolyzer: 2019-08-05 update knowledgebase
Open Targets: release of the Platform - 22.11, 24 November 2022
AutDB : Updated Sep, 2022

## SUPPLEMENTARY FIGURES

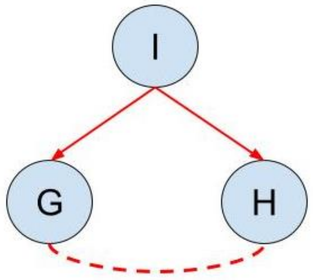


**Supplementary Figure 1. Dependency model**: A graphical model where entities represented as nodes: affected individuals (I), phenotypes (H), and genes (G) are connected with edges with respective directionality representing their underlying relationship. The affected individuals have phenotypes and carry genetic variants hence have direct edges. The relationship between genetic variants and corresponding phenotypes are inferred conditionally with respect to these individuals.

**Supplementary Figure 2**. **Cluster level analysis of the hierarchical clustering results.** Hierarchical agglomerative clustering resulted in 16 clusters, where each cluster showed a dominant co-occurring phenotype of the GDD. For the analysis of each cluster, we plotted four different properties in a row for each of the 16 clusters. In supplementary Figure 3, histograms in first column shows the top 5 phenotypes of the clusters, second column shows the Silhouette cluster validation index, third column shows the frequency of number of phenotypes individuals have, and fourth column represent the frequency of shared phenotypes among individuals.

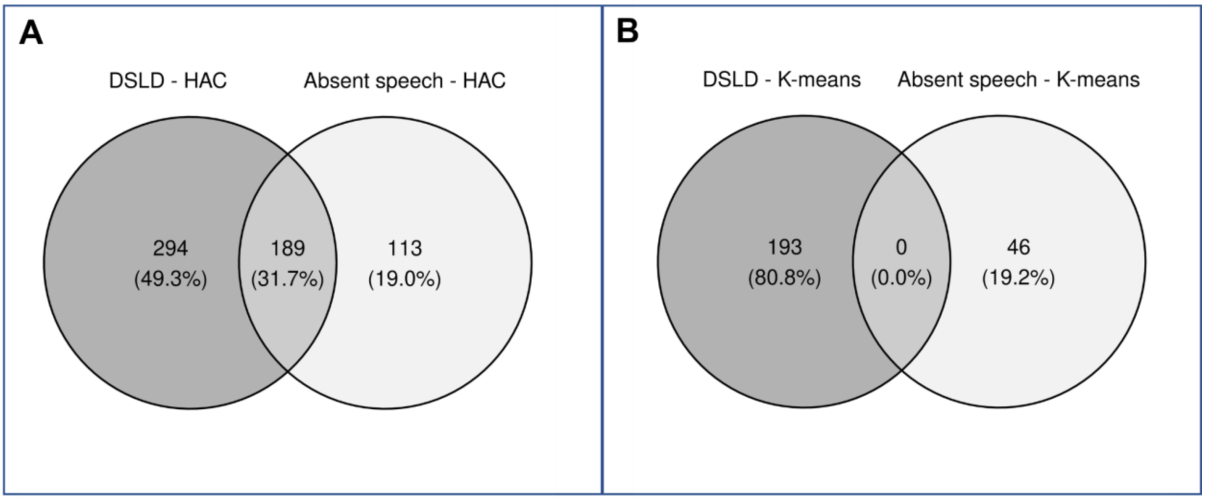


**Supplementary Figure 3. Overlap between genes found in individuals with delayed speech and language development (DSLD) vs absent speech (AS). A)** Using HAC, 31% of genes overlapped between DSLD and AS. Overlapping genes are involved in: a) no enriched molecular function but reactome identifies cilium assembly and visual phototransduction (FDR: 1.52e-10 and 0.00083), b) DNA binding (FDR 1.78e-07), and c) catalytic activity (1.36e-07). DSLD specific genes were involved in: a) protein and transcription factor binding (FDR: 5.12e-05 and 0.00076), b) catalytic activity acting on ARNt and ARN (FDR: 7.11e-21 and 4.01e-20), and c) mitochondria-associated terms and as a molecular function, oxidoreductase (FDR: 0.00051). Genes specific to AS had a more dispersed and less interconnected network with distinct sub-clusters with no enriched molecular function but biological processes and cellular components related to synapses and ion channel complexes and that interaction between L1 and ankyrins and L1CAM interactions are enriched in the reactome (FDR : 0.0099) (see **Supp. Table 6** for the full list of genes). **B)** K-means clustering led to very distinct gene groups with no overlap.

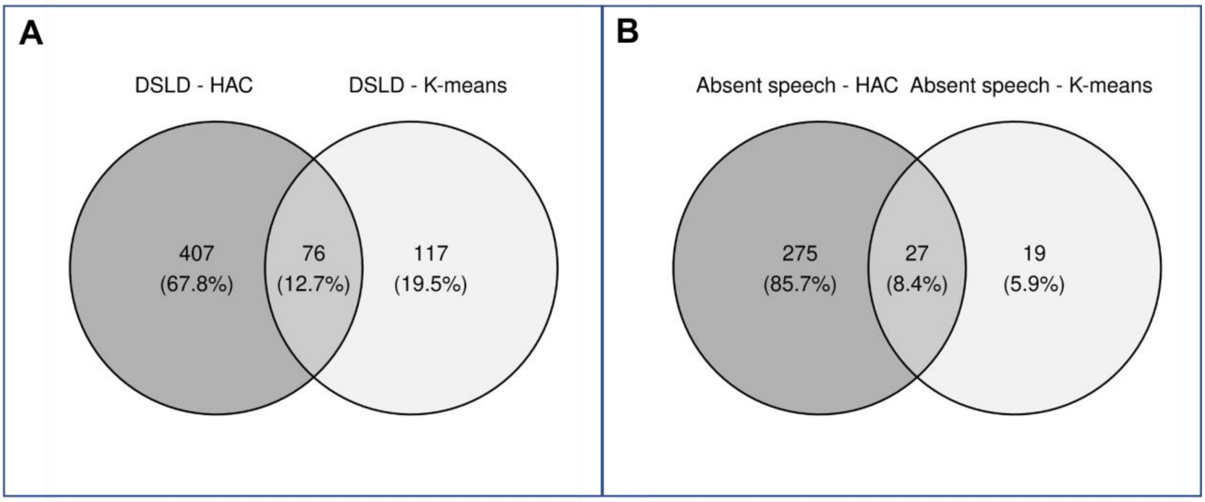


**Supplementary Figure 4. Overlap between genes found by HAC and k-means clustering. A)** Genes identified for delayed speech and language development (DSLD). **B)** Genes identified for absent speech (AS).

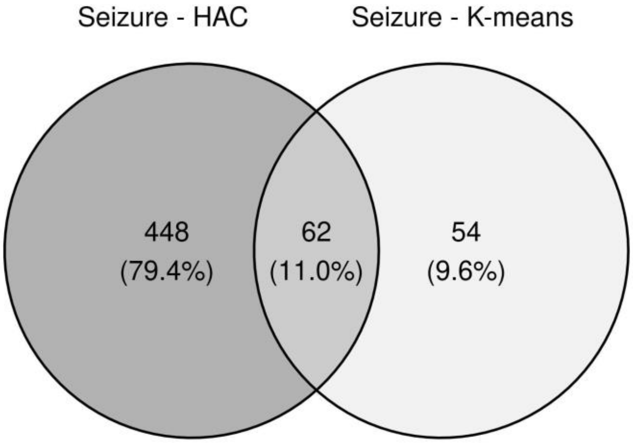


**Supplementary Figure 5. Venn diagram representing genes identified by HAC versus k-means clustering for individuals with GDD and seizures.** Genes identified via k-means were present either as unique (9.6%) or overlapping with those identified via HAC (11%). HAC on the other hand identified most genes as unique (79%).

## SUPPLEMENTARY TABLES

**Supplementary Table 1.** Curated Gene list of GDD and ID from the literature and genotypic diversity in GDD.

**Supplementary Table 2: Breakdown of specific seizure types present in DDD**. The phenotypes are listed as available from DDD using HPO terms. Some HPO terms represent related entities (for instance, febrile seizure, complex febrile seizure, and simple febrile seizure) and are therefore presented in the manuscript following concatenation of several HPO terms. This illustrates that HPO terms can be very specific and that in some cases the annotator did not use the most specific term either by choice or because the clinical information did not allow it potentially.

**Supplementary Table 3. Morphological and physical findings found most commonly in individuals with global developmental delay (GDD) (>1%).** We present here the dysmorphic features, malformations, or other anatomical phenotypes as opposed to the behavioral and functional ones.

**Supplementary Table 4. Network and subnetwork enrichment of cluster 4 (DSLD) from HAC.** Sheet 1 - MCL clustering results from the gene network of individuals in Cluster 4, yielding 27 sub-networks. 12 of these sub-networks contain more than 10 genes. Sheets 2-13 - Enrichment analysis results for the 12 sub-networks with more than 10 genes in Cluster 4 (DSLD) from the MCL clustering of the gene network.

**Supplementary Table 5. Network and subnetwork enrichment of cluster 14 (Absent speech) from HAC.** Sheet 1 - MCL clustering results from the gene network of individuals in Cluster 14, yielding 23 sub-networks. 8 of these sub-networks contain more than 10 genes. Sheets 2-9 - Enrichment analysis results for the 8 sub-networks with more than 10 genes in Cluster 4 (DSLD) from the MCL clustering of the gene network.

**Supplementary Table 6. Network and subnetwork enrichment of specific and shared genes of cluster 4 & cluster 14 from HAC.** Sheet 1 - MCL clustering results from the gene network of specific genes of cluster 4, yielding 27 sub-networks. Sheets 2-4 - Enrichment analysis results for the 3 sub-networks with enrichment results from the MCL clustering of specific genes of cluster 4. Sheet 5 - MCL clustering results from the gene network of shared genes of cluster 4 & 14, yielding 16 sub-networks. Sheets 6-8 - Enrichment analysis results for the 3 sub-networks with enrichment results from the MCL clustering of shared genes of cluster 4 &14. Sheet 9 - MCL clustering results from the gene network of specific genes of cluster 14, yielding 16 sub-networks. Sheets 10-12 - Enrichment analysis results for the 3 sub-networks with enrichment results from the MCL clustering of specific genes of cluster 14.

**Supplementary Table 7. Network and subnetwork enrichment of specific genes for DSLD from k-means.** Sheet 1 - MCL clustering results from the gene network of specific genes for DSLD from k-means, yielding 24 sub-networks. 3 of these sub-networks contain more than 10 genes. Sheets 2-4 - Enrichment analysis results for the 3 sub-networks with more than 10 genes from the MCL clustering of the gene network.

**Supplementary Table 8. Network and subnetwork enrichment of specific genes for AS from k-means.** Sheet 1 - MCL clustering results from the gene network of specific genes for AS from k-means, yielding 5 sub-networks. 1 of these sub-networks contain more than 10 genes. Sheets 2-4 - Enrichment analysis results for the 3 sub-networks with enrichment results from the MCL clustering of the gene network.

**Supplementary Table 9. Network and subnetwork enrichment of cluster 2 (Seizure) from HAC.** Sheet 1 - MCL clustering results from the gene network of individuals in Cluster 2, yielding 30 sub-networks. 13 of these sub-networks contain more than 10 genes. Sheets 2-14 - Enrichment analysis results for the 13 sub-networks with more than 10 genes in Cluster 4 (DSLD) from the MCL clustering of the gene network.

**Supplementary Table 10. Network and subnetwork for GDD and seizure from K-means.** Sheet 1 - MCL clustering results from the gene network of specific genes for seizure from k-means, yielding 14 sub-networks. 2 of these sub-networks contain more than 10 genes. Sheets 2-4 - Enrichment analysis results for the 3 first sub-networks from the MCL clustering of the gene network.
