# Supplementary figures and images for: Developing a cluster-based approach for deciphering complexity in individuals with neurodevelopmental differences
Source: Front Pediatr. 2023 Sep 18;11:1171920. doi: 10.3389/fped.2023.1171920 (PMC10543689; doi:10.3389/fped.2023.1171920)

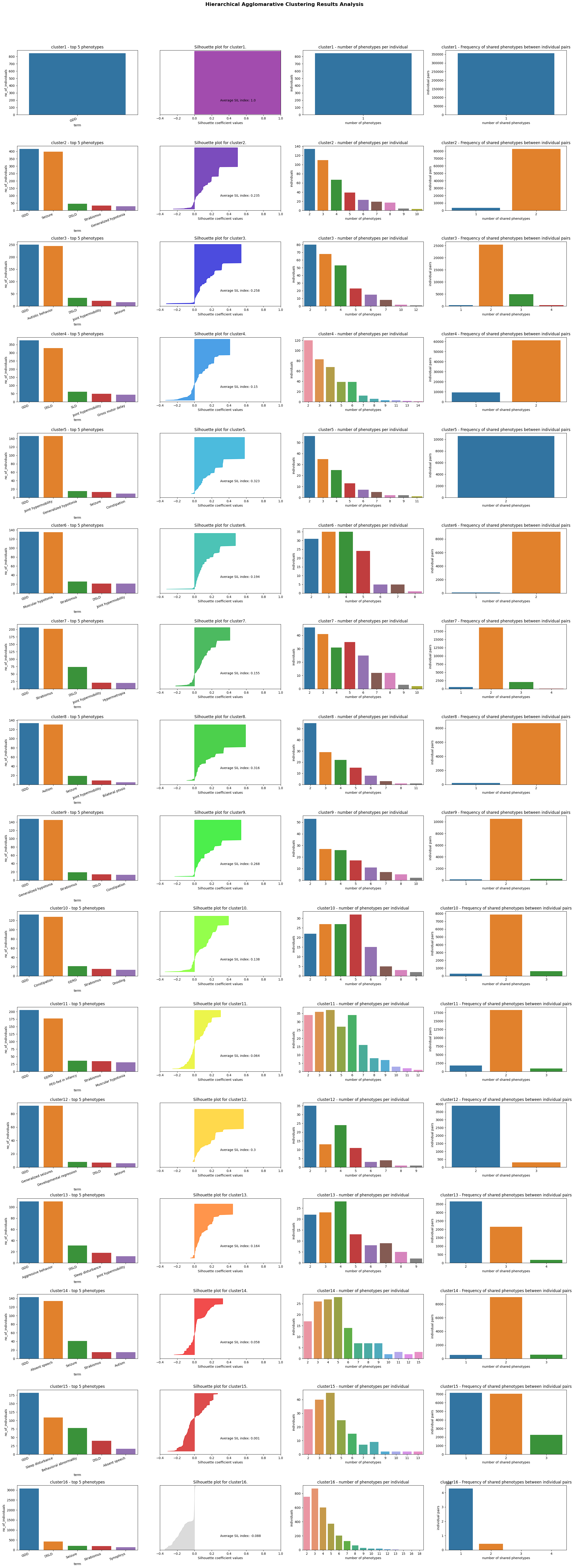

Supplement: Supplementary file 12 [file Image1.png]
